# Cell Cycle Dependent Association of EBP50 with Protein Phosphatase 2A in Endothelial Cells

**DOI:** 10.1371/journal.pone.0035595

**Published:** 2012-04-16

**Authors:** Anita Boratkó, Pál Gergely, Csilla Csortos

**Affiliations:** 1 Department of Medical Chemistry, University of Debrecen Medical and Health Science Center, Debrecen, Hungary; 2 Cell Biology and Signaling Research Group of the Hungarian Academy of Sciences, University of Debrecen Medical and Health Science Center, Debrecen, Hungary; Hungarian Academy of Sciences, Hungary

## Abstract

Ezrin-radixin-moesin (ERM)-binding phosphoprotein 50 (EBP50) is a phosphorylatable PDZ domain-containing adaptor protein that is abundantly expressed in epithelium but was not yet studied in the endothelium. We report unusual nuclear localization of EBP50 in bovine pulmonary artery endothelial cells (BPAEC). Immunofluorescent staining and cellular fractionation demonstrated that EBP50 is present in the nuclear and perinuclear region in interphase cells. In the prophase of mitosis EBP50 redistributes to the cytoplasmic region in a phosphorylation dependent manner and during mitosis EBP50 co-localizes with protein phosphatase 2A (PP2A). Furthermore, *in vitro* wound healing of BPAEC expressing phospho-mimic mutant of EBP50 was accelerated indicating that EBP50 is involved in the regulation of the cell division. Cell cycle dependent specific interactions were detected between EBP50 and the subunits of PP2A (A, C, and Bα) with immunoprecipitation and pull-down experiments. The interaction of EBP50 with the Bα containing form of PP2A suggests that this holoenzyme of PP2A can be responsible for the dephosphorylation of EBP50 in cytokinesis. Moreover, the results underline the significance of EBP50 in cell division via reversible phosphorylation of the protein with cyclin dependent kinase and PP2A in normal cells.

## Introduction

Ezrin-radixin-moesin (ERM) binding phosphoprotein of 50 kD (EBP50) is a member of the Na^+^/H^+^ exchanger regulatory factor (NHERF) family which consists of four related PDZ (postsynaptic density 95/discs-large/zona occludens-1) domain containing scaffolding proteins termed as NHERF1/EBP50, NHERF2/E3KARP, NHERF3/PDZK1, and NHERF4/IKEPP [1]. NHERF1 was originally recognized as Na^+^/H^+^ exchanger-3 binding partner [2], and it has later been identified as an ERM binding phosphoprotein [3]. NHERFs are highly abundant in the epithelium and their role in Na^+^/H^+^ exchanger-3 regulation is well established [4], therefore EBP50 was characterized mainly in polarized epithelial cells up to the present.

EBP50 has two PDZ domains and a C-terminal ERM-binding domain. It is believed that through these domains EBP50 forms bridges among plasma-membrane and cytoskeleton proteins. Its function, for example, in microvillar assembly as part of the PDZK1/NHERF3-EBP50-ezrin complex was studied in details [5]. It seems that EBP50 binds ezrin specifically in epithelial cells and there is an interdependence of EBP50 and ezrin for their apical localization [6], [7]. However, recent paper describes the effect of EBP50-moesin interaction in the contractile response of artery [8]. Most of the interacting proteins bind to the first PDZ domain, only a few partners relate with the second PDZ, like beta-catenin [9]. Self association of EBP50 through the PDZ domains [10], and the intramolecular interactions of EBP50 between the PDZ2 and C-terminal domains result in an autoinhibition of complex formation [11]. Protein-protein interactions between the members of the NHERF family [5], [12] have been described, as well.

EBP50 is a subject to phosphorylation by several kinases and these modifications have been suggested to alter its binding activity. Oligomerization of EBP50 was shown to be regulated via site-specific phosphorylation. Phosphorylation by PKC on Ser^337^/Ser^338^ enhances the oligomerization [13]; and G protein-coupled receptor kinase 6 was identified as the kinase responsible for constitutive phosphorylation of Ser^289^ which facilitates PDZ domain mediated interactions [12], [14]. During mitosis EBP50 is phosphorylated on Ser^279^ and Ser^301^ by the cyclin dependent kinase 1 (Cdk1) and that phosphorylation inhibits its oligomerization, but allows association with Pin1, a peptidylprolyl isomerase [15]. In addition, it was shown by S77A and S77D substitutions that phosphorylation of the PDZ1 domain attenuates co-localization of EBP50 with the cortical actin [16]. In agreement with the outcome of phosphorylation on oligomerization, it was demonstrated that PKC activation and EBP50 phosphorylation promotes microvili rearrangement [17]. Recent study also showed that phosphorylation of EBP50 by PKC within the PDZ2 domain reduced its association with the cystic fibrosis transmembrane conductance regulator [18]. On the other hand, phosphorylation of EBP50 by Cdk1 inhibits its role in microvili formation in interphase but not in mitotic cells [17].

Dephosphorylation of the above mentioned sites is an equally important element of the reversible phosphorylation, however, phosphatases specific for EBP50 have not been identified yet. Protein phosphatase 1 (PP1), 2A (PP2A), and 2B (PP2B) are the major classes of serine/threonine specific protein phosphatases, each having a heterodimer or –trimer holoenzyme form of one of the catalytic subunits and one or two of the large number of the variable regulatory subunits [19].

The aim of the present work was to characterize localization, phosphorylation/dephosphorylation of the ERM binding phosphoprotein, EBP50, in bovine pulmonary artery endothelial cells (BPAEC). We found mainly nuclear localization of EBP50 in interphase cells, and its redistribution to the cytoplasm during the course of mitosis parallel with its phosphorylation. PP2A was identified as an interacting protein of EBP50 during pro-, prometa-, meta-, ana-, telophase, and early cytokinesis supporting that PP2A is responsible for the dephosphorylation of the Cdk1 phosphorylation sites.

## Results

### Nuclear localization of EBP50 in endothelial cells

**Figure 1:**
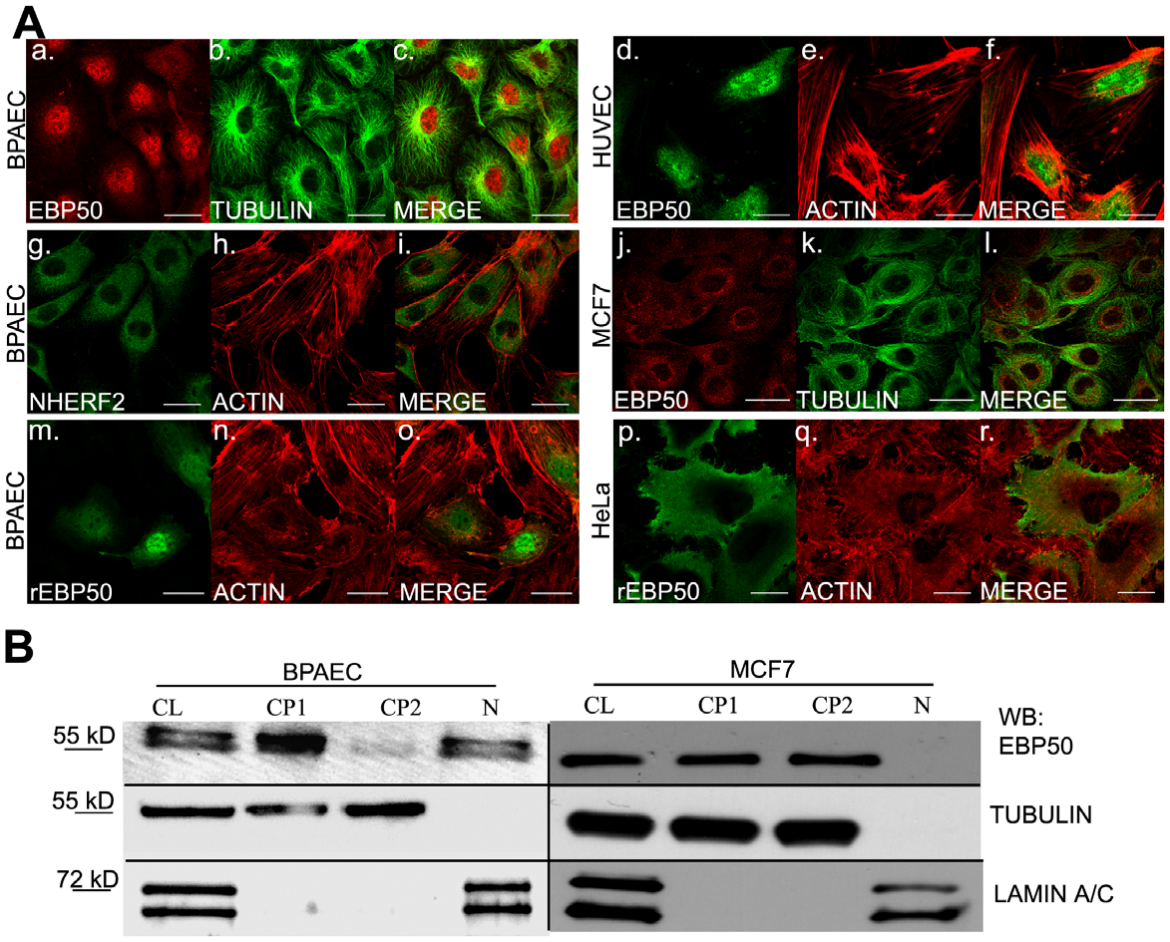
Nuclear localization of EBP50 in BPAEC. *A)* Immunofluorescence staining of confluent BPAEC (*a–c,g–i*), HUVEC (d–f), MCF7 (*j–l*) and pCMV-myc EBP50 transfected BPAEC (*m–o*), and HeLa (*p–r*) cells was performed using anti-EBP50 (anti-SLC9A3R1 antibody, Abgent) (*a,j:* red, *d:* green), anti-tubulin (*b,k:* green), anti-NHERF2 (*g:* green), and monoclonal anti-c-myc (*m,p:* green) primary antibodies. Actin microfilaments were stained with Texas Red conjugated phalloidin (*e,h, n,q:* red). *c,f,i,l,o* and *r* are merged images of *a–b, d–e, g–h, j–k, m–n, and p–q,* respectively. Representative data of at least three independent experiments are shown. Scale bars: 100 µm. *B)* Cellular fractionations of BPAE and MCF7 cells were made as described in Materials and Methods. The fractions were analyzed with anti-EBP50 (anti-SLC9A3R1 antibody Abgent), anti-β-tubulin as a cytoplasmic and anti-lamin A/C antibodies as a nuclear marker. CL: cell lysate, CP1: cytoplasmic fraction 1, CP2: cytoplasmic fraction 2, N: nuclear fraction.

To study subcellular localization of EBP50 in endothelial cells (EC), we co-stained BPAEC with anti-EBP50 and anti-β-tubulin antibody. By confocal microscopy EBP50 was detected mainly in the nucleus and in the perinuclear region (Fig. 1A.a–c and Fig. S1, parallel experiments using two different anti- EBP50 antibodies). In a parallel experiment we also found EBP50 in the nuclei of HUVEC (Fig. 1A.d–f), but in the cytoplasm of MCF7 human breast adenocarcinoma cell line (Fig. 1A.j–l). Because EBP50 shares high sequence homology with NHERF2, to exclude the possibility of cross-reaction of the EBP50-specific antibody with NHERF2, we stained BPAEC with NHERF2 specific antibody as well. Our result clearly shows that NHERF2 localizes in the cytoplasm but not in the nucleus (Fig. 1A.g–i).

To ensure this rather unique nuclear appearance of EBP50 in EC we cloned bovine EBP50 into a pCMV-Myc plasmid and transfected the construct into endothelial and epithelial cells. Immunofluorescent staining with tag specific antibody showed the same result as above, namely, the recombinant EBP50 was in the nuclear and perinuclear region in EC (Fig. 1A.m–o), however, it was present in the cytoplasm of the epithelial cells (Fig. 1A.p–r).

To further strengthen our observation subcellular fractionations of BPAEC and MCF7 were performed. Western blot analysis of the fractions proved that EBP50 is present in the nuclear fraction only in the endothelial cells. The purity of fractions was confirmed using anti-β-tubulin antibody as a cytoplasmic and anti-lamin A/C antibody as a nuclear marker (Fig. 1B). Moreover, the nuclear appearance of EBP50 in endothelial cells was detected by immunohistochemical study (Fig. S2).

### Phosphorylation dependent localization of EBP50

**Figure 2:**
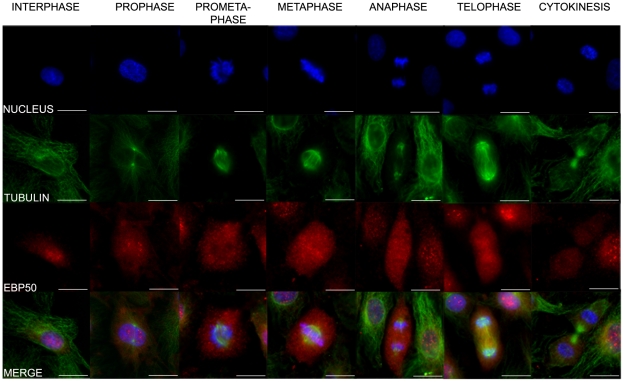
Localization of EBP50 during the phases of the cell cycle. Immunofluorescence staining of BPAE cells was performed using anti-EBP50 (red) primary antibody (anti-SLC9A3R1 antibody, Abgent). Phases of the cell cycle were identified by tubulin (green) and DAPI (blue) staining. Representative images from five independent experiments are shown. Scale bar: 100 µm.

Although the nuclear appearance of EBP50 was quite uniform for the EC monolayer studied, we also observed that EBP50 left the nuclear region in dividing cells. To identify the time-point of re-localization we co-stained BPAEC with anti-EBP50 and anti-β-tubulin antibodies, and DAPI. EBP50 was in the nucleus in interphase cells, however, during prophase its redistribution to the cytoplasm was apparent and we could detect its disappearance from the cytosol only in the phase of cytokinesis (Fig. 2).

**Figure 3:**
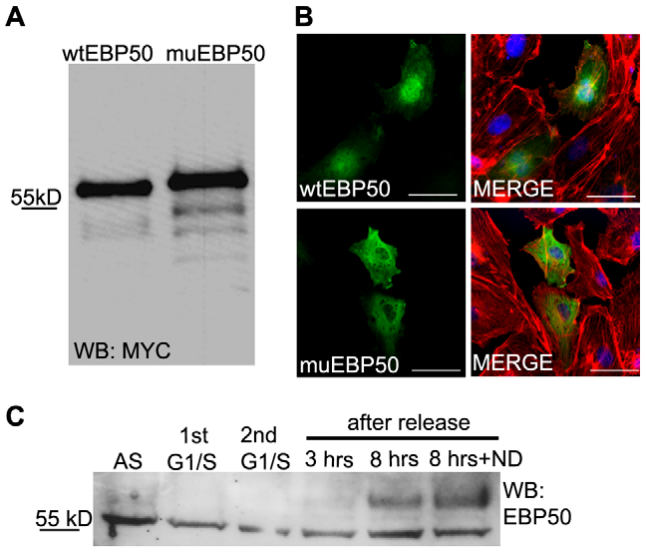
Localization of EBP50 is phosphorylation-dependent in BPAEC. pCMV-Myc EBP50 wild type (wtEBP50) and pCMV-Myc EBP50 S288D∶S310D phosphomimic mutant (muEBP50) proteins were analysed by Western blot *(A)* and immunofluorescence *(B)*. Anti-myc (green) antibody was used for labeling of wild type and mutant EBP50 both in Western blot and immunofluorescent experiments. Actin microfilaments were stained with Texas Red conjugated phalloidin (red) and DAPI (blue) staining was used to visualize the nuclei. Scale bar: 100 µm. Panel *C*: Phosphorylation level of EBP50 in synchronized BPAE cells. Cells were arrested in G1/S phase using double thymidine block and in mitotic phase by nocodazole treatment as described in Materials and Methods. Samples of asynchronized cells (AS), 1st and 2nd thymidine block cells (G1/S), and cells after 3 or 8 h release of the thymidine block without or with addition of 80 ng/ml nocodazole (ND) were tested for EBP50 (anti-NHERF1(A310) antibody, Cell Signaling Technology) by Western blot. Representative images from at least three independent experiments are shown.

Cdk1 catalyzed phosphorylation and mobility-shift upon SDS-PAGE of EBP50 in mitotic cells were described in HeLa cells [15]. Therefore, we hypothesized that the varying localization of the protein during the course of mitosis can be related to its phosphorylation state. To prove our hypothesis first a phospho-mimic construct of EBP50 was created by mutation of Ser288 and Ser310 side-chains, which were known to be phosphorylated in mitotic cells by Cdk1 [15], to Asp to imitate phosphorylation. On SDS-PAGE the apparent size of EBP50 mutant shifted upward compared to the recombinant wild type EBP50 (Fig. 3A). Next we confirmed by immunofluorescent staining that the phospho-mimic mutant form of EBP50 showed cytoplasmic allocation but the wild-type recombinant protein was in the nucleus (Fig. 3B).

To verify the phosphorylation of the endogenous EBP50 we synchronized BPAEC. Cells were arrested in G1/S using double thymidine block, and G2/M phase arrest was established using nocodazole treatment. EBP50 was detected as a 55 kD band in the lysate of asynchronized cells, however, we observed an additional pale band at higher molecular mass that completely disappeared in the lysate of cells in G1/S. Conversely, the band of higher mass had a massive manifestation 8 h after releasing the cells from the thymidine block, as the majority of cells got near to the phase of mitosis (Fig. 3C). Based on the previously published data [15] and our results (Fig. 3A–B) this higher molecular mass band could be the phosphorylated form of EBP50. Furthermore, these observations proved our hypothesis that the relocalization of EBP50 occurs in a phosphorylation-dependent manner.

### Phospho-mimic EBP50 supports wound healing

**Figure 4:**
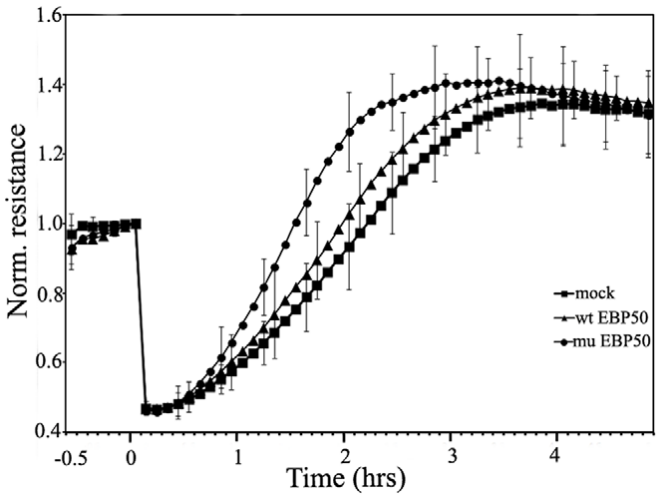
Phospho-mimic EBP50 supports wound healing. BPAEC were transfected with pCMV-Myc EBP50 wild type (wt EBP50) and pCMV-Myc EBP50 S288D∶S310D phosphomimic mutant (mu EBP50) and were plated onto two 8W10E arrays 24 h post-transfection. After cells achieved monolayer density (about 1000 Ω impedance) an alternate current of 5 mA at 60 kHz frequency was applied for 30 sec duration to establish wounds in the cell layer (0 h); after that the impedance was measured for 5 h. The average results from three independent experiments each made with two or three parallel measurements are shown. Error bars represent SD.

As proliferation/cell division and migration are essential in wound healing [20], [21], to further demonstrate the significance of the phosphorylation of EBP50 during the cell cycle, we compared the timing of wound healing of mock-transfected BPAEC monolayer with the ones overexpressing wild type- or phospho-mimic EBP50 using electric cell impedance measurements, ECIS (Fig. 4). The time from the beginning of wound healing to the time at 50% of the maximum impedance (effective time) was about the same for the mock-transfected control (1.9 h) and the sample overexpressing the wild type EBP50 (1.8 h). However, the phopho-mimic mutant form of EBP50 significantly accelerated the healing process (effective time = 1.5 h).

### EBP50 associates with PP2A-Bα holoenzyme

As protein phosphorylation/dephosphorylation is a reversible process, next we intended to identify the protein phosphatase catalyzing the dephosphorylation of EBP50. First BPAEC were arrested in mitotic phase and at the point of the release the media were implemented with specific protein phosphatase inhibitors. Okadaic acid of 5 nM was able to maintain the phosphorylation state of EBP50 for 6 h after the release suggesting that PP2A is involved in the dephosphorylation. Furthermore, 2 nM calyculin A, a specific inhibitor of PP1, had no effect on the above dephosphorylation process (unpublished data).

**Figure 5:**
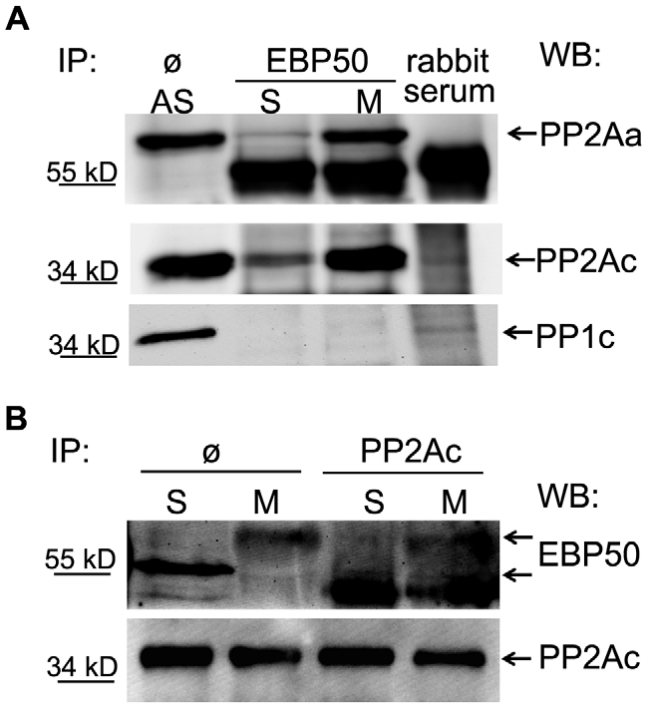
PP2A subunits associate with cellular EBP50. EBP50 (panel *A*) or PP2A C subunit (panel *B*) was immunoprecipitated from lysates of thymidine or nocodazole treated BPAEC as described in Materials and Methods. The IP complexes were probed for PP2Aa, PP2Ac, and PP1c (α and δ) (*A*) or EBP50 (anti-NHERF1(A310) antibody, Cell Signaling Technology) and PP2Ac subunit (*B*). Ø: cell lysate without immunoprecipitation, AS: asynchonized cell lysate, S: early S phase cell lysate, M: mitotic phase lysate. Additional bands at 55 kD are IgG. Representative blots from three independent experiments are shown.

Subsequently, we examined whether protein-protein interactions could be detected between EBP50 and PP2A or PP1. Lysates from thymidine or nocodazole treated cells were prepared and the endogenous EBP50 was immunoprecipitated from each lysate using anti-EBP50 antibody. The immunoprecipitated complexes were probed in Western blot with antibodies raised against different protein phosphatase subunits. As shown in Figure 5A, EBP50 co-immunoprecipitated with the A (PP2Aa) and C (PP2Ac) subunits of PP2A, but no interaction of EBP50 was detected with PP1c α and δ isoforms. EBP50 was also observed in PP2Ac immunoprecipitates of lysates with thymidine or nocodazole treatments. The interaction between EBP50 and PP2A was clearly more pronounced in lysates of mitotic phase cells (Fig. 5B).

**Figure 6:**
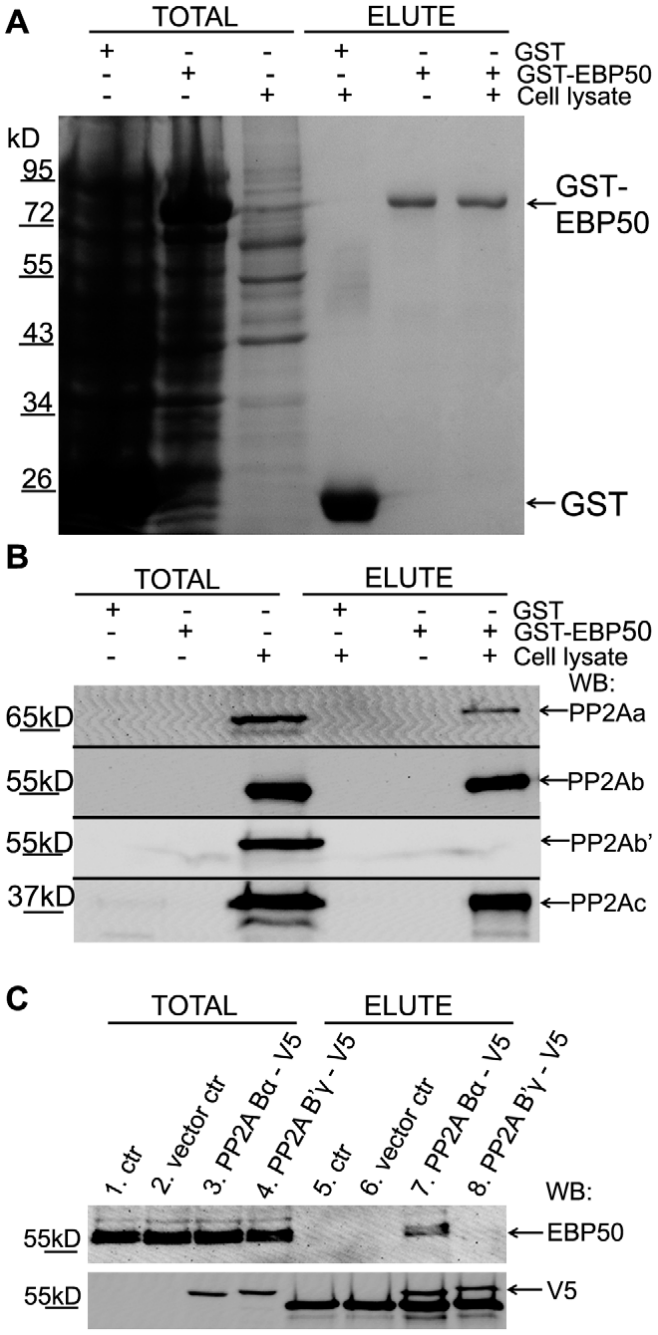
Bα subunit of PP2A interacts with EBP50. Panels *A* and *B*: Bacterially expressed glutathione S-transferase (GST) and GST-tagged wild-type EBP50 were loaded onto glutathione-Sepharose as described in Materials and Methods. After a washing step the resin samples were incubated with BPAEC lysate or cell lysis buffer. Non-binding proteins were washed out and the bound proteins were eluted with 10 mM glutathion. Coomassie staining *(A)* and Western blot probed with PP2A A, B, B′, or C specific antibodies *(B)* of the bacterial and endothelial cell lysates (Total) and the eluted fractions after the pull-down are shown. Panel *C*: BPAEC monolayers were transfected with pcDNA3.1 V5-His (vector ctr), pcDNA3.1 V5-His PP2A Bα (PP2A Bα–V5) and pcDNA3.1 V5-His B′γ (PP2A B′γ–V5) plasmids. Lysates of the transfected cells were incubated with Anti-V5 Agarose Affinity Gel as described in Materials and Methods, and the bound proteins were eluted by boiling the resin in 1× SDS sample buffer. Western blot analysis of the lysates of transfected cells (Total) and eluted samples were done using EBP50 (upper panel) and V5-tag (lower panel) specific antibodies. Additional bands at 55 kD are IgG. Representative data of three independent experiments are shown.

Since the apparent size of the B subunits (about 55 kD) is close the size of IgG we could not detect co-immunoprecipitation of the B subunits with EBP50. To identify the third, variable B subunit of PP2A - which determine the substrate specificity and subcellular localization of the holoenzyme – GST-tagged bacterial expression construct of EBP50 was created. Immobilized recombinant EBP50 or GST protein, as a negative control, were incubated with or without BPAEC lysates in pull down experiments. The total cell lysates and the eluted proteins were analyzed by Coomassie staining (Fig. 6A) and by Western blot (Fig. 6B) using antibodies for different PP2A subunits. The Coomassie staining shows the efficiency of the purification from the bacterial extract. The results of the Western blot indicate the presence of B subunit in the GST-EBP50 pull-down along with A and C subunits of PP2A (Fig. 6B). In the same set of experiment B′ subunit of PP2A was not detectable.

We were able to confirm the specific interaction between EBP50 and PP2A Bα subunit using overexpressed B subunits with Anti-V5 Affinity Gel. BPAEC were transfected with expression contructs containing the coding sequence of PP2A Bα or B′γ subunits and the overexpressed proteins were bound to the anti-V5 antibody immobilized on the resin. The eluted recombinants and the presence of EBP50 in the eluted fractions were analyzed by Western blot (Fig. 6C). Although the amount of Bα and B′γ subunits appears the same (lower panel), the binding of endogenous EBP50 was detectable only to Bα. Taken together, data of immunoprecipitations demonstrate that endogenous EBP50 and endogenous PP2A physically associate, moreover, our results indicate that EBP50 interacts with the holoenzyme form of PP2A containing Bα.

**Figure 7:**
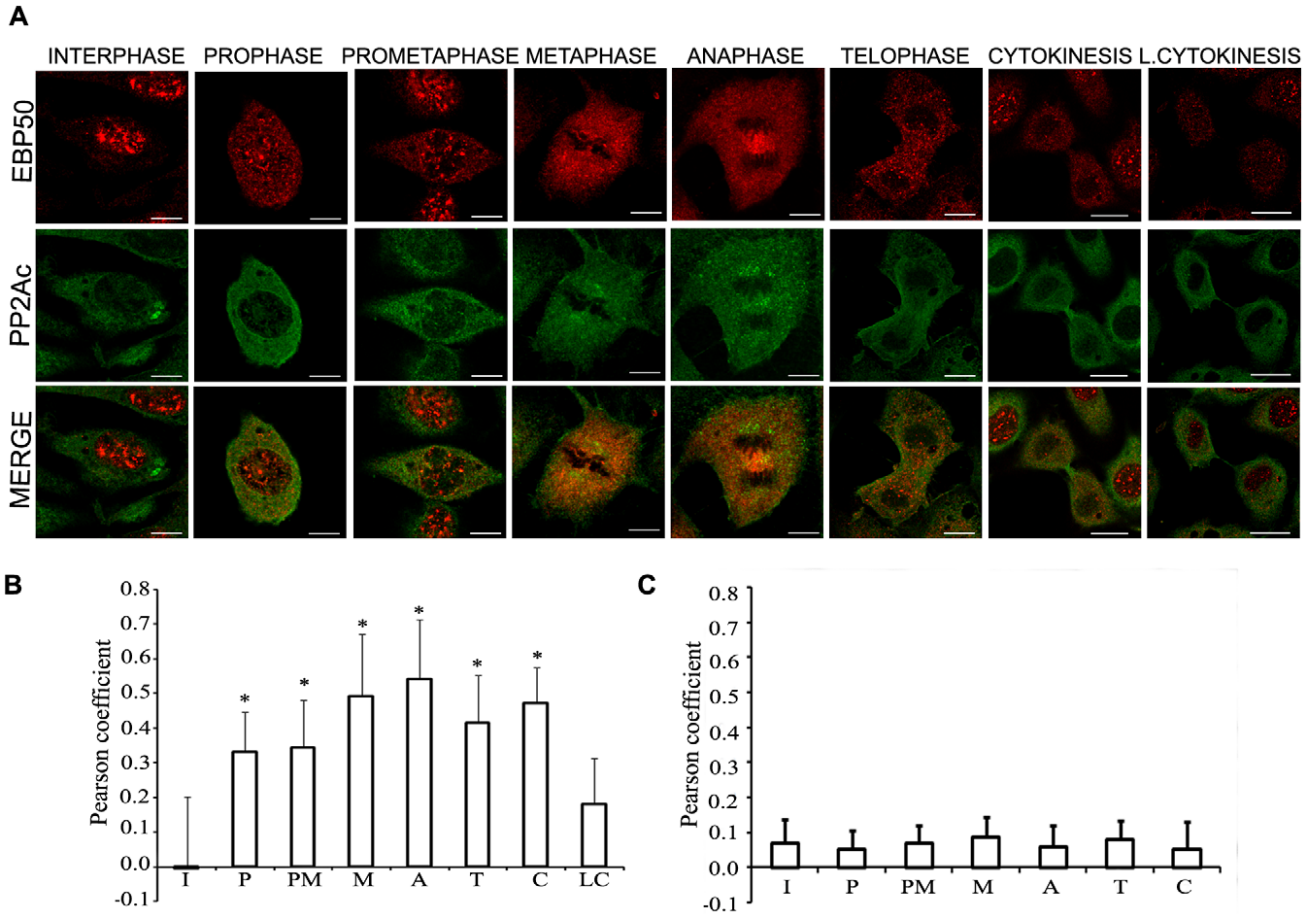
Co-localization of EBP50 and PP2Ac during mitosis in BPAEC. *(A)* Immunofluorescence staining of BPAEC was performed using anti-EBP50 (anti-SLC9A3R1 antibody, Abgent) (red) and anti-PP2Ac (green) primary antibodies. Phases of the cell cycle were identified using DAPI staining (not shown). Scale bars: 100 µm. Co-localization of EBP50 *(B)* or NHERF2 *(C)* and PP2Ac was evaluated by determination of Pearson cross-correlation coefficient. The results are presented as means ± SD from 50–100 *(B)* or 25–30 *(C)* independent cells for each studied phase of the cell cycle. Statistical analysis was done with ANOVA on ranks. Significant changes compared to the interphase cells are indicated by * (P<0.05). I: interphase, P: prophase, PM: prometaphase, M: metaphase, A: anaphase, T: telophase, C: cytokinesis, LC: late cytokinesis.

The mitotic phase interaction led us to search possible co-localization of EBP50 with PP2A during mitosis. Therefore, BPAEC were co-stained with anti-EBP50 and anti-PP2Ac antibodies (Fig. 7A). While EBP50 has nuclear and perinuclear distribution in interphase cells, PP2Ac appears to be present mainly in the cytoplasm, consequently, merge of parallel images emphasizes no overlap of staining. However, in agreement with our immunoprecipitation experiments which indicated strong protein-protein interaction between EBP50 and PP2A, we detected co-localization of EBP50 and PP2Ac in pro-, prometa-, meta-, ana-, and telophase. We also observed that the two proteins still co-localize at the beginning of cytokinesis, when EBP50 showed cytoplasmic appearance. On the other hand, no co-localization could be observed in the late cytokinesis. To quantify the extent of association the Pearson's correlation coefficient was determined at the different phases of the cell cycle. Positive value of the coefficient indicates co-localization/association of the studied proteins. Significant co-localization was found from the prophase till the early cytokinesis, but not in late cytokinesis, compared to interphase (Fig. 7B).

To confirm that the EBP50-PP2A interaction is specific and phosphorylation dependent, the same analysis of cells co-stained with PP2Ac and NHERF2 specific primary antibodies was performed. NHERF2 has the highest amino acid sequence homology with EBP50 in the NHERF family, but NHERF2 does not exhibit possible Cdk1 phosphorylation sites. Although both PP2Ac and NHERF2 are in the cytoplasm of interphase cells, neither co-localization nor interaction between these proteins in any phase of the observed cells could be detected (Fig. 7C).

## Discussion

EBP50 is a well characterized protein in epithelium, yet little is known about its function in other cell types. It is expressed at high levels in the kidney, liver, small intestine, and placenta, but also expressed at lower levels in the brain and lung [2], [3]. Based on studies of epithelial cells where EBP50 is localized to the plasma membrane and the cytoplasm, it is accepted that its primary function is to act as a scaffold protein linking transmembrane proteins to various cytoskeletal proteins. In polarized cells such as kidney proximal tubule cells it is localized primarily in the apical region [22] where its association with proteins like cystic fibrosis transmembrane conductance regulator, podocalyxin, G-protein coupled- or tyrosine kinase receptors was found [23], [24], [25].

In interphase BPAEC and HUVEC we detected EBP50 in the nucleus and in the perinuclear region in contrast with the above mentioned cytoplasmic location found by others in epithelial cells. Our observation was verified by immunfluorescent staining, cell fractionation, and recombinant protein expression. The amino acid sequence deduced from the DNA coding the endothelial EBP50 (SLC9A3R1) is identical with the published bovine sequence in the database (NM_001077852). Therefore the varying location of EBP50 in endothelial and epithelial cells may be the consequence of different interactive protein partners suggesting different function of EBP50 in the two cell types. We also compared the localization of EBP50 and NHERF2 in BPAEC, as these proteins are the most similar members of the NHERF family (49% amino acid identity). Results of immunoflurescence clearly excluded the possibility of non-specific binding of the primary antibodies; EBP50 and NHERF2 appeared at different locations in BPAEC. In addition, we detected the same pattern of localization of the recombinant EBP50, as well. Only a few studies [26], [27] have reported nuclear localization of EBP50 but only in cancer cell lines not in normal cells. It was shown that EBP50 is overexpressed in hepatocellular carcinoma cell lines and it is localized in the cytoplasm and in the nucleus [9]. In addition, cell cycle dependent shuttling between the nucleus and the cytoplasm of merlin, an ERM-like tumor suppressor protein, was described [28] in glioma and osteosarcoma cells. Interestingly, others [29] also detected ERM proteins in the nucleus of MDCK and HeLa cells.

It is known that EBP50 can be phoshorylated by protein kinases on multiple sites. It is phosphorylated during mitosis by Cdk1 at Ser^279^ and Ser^301^ and this phosphorylation inhibits its oligomerization [15]. The SPX(K/R) preferred phosphorylation motifs of Cdk1 are conserved in EBP50 of different species, thus Ser^279^ and Ser^301^ residues in the rabbit protein referred in [15] correspond to Ser^288^ and Ser^310^ in the bovine EBP50. Therefore we concluded that the cell cycle dependent localization change and mobility shift on SDS-PAGE of the endothelial EBP50 we observed are in parallel with the phosphorylation and possibly the oligomerization state of the protein. Furthermore, phosphorylation of EBP50 by the employment of its phospho-mimic mutant (S288D∶S310D) form showed mobility shift on SDS-PAGE and cytoplasmic localization as did the endogenous EBP50 in dividing cells.

The nuclear envelope offers a selective barrier between the nuclear compartment and the cytoplasm. At the transition from prophase to prometaphase of mitosis the breakdown of the nuclear envelope leads to combination of the two compartments [30]. However, we could detect EBP50 in the cytoplasm in cells in early prophase to late cytokinesis, suggesting an active transporting mechanism. A number of proteins required for nucleocytoplasmic transport have important functions during mitosis [31], still the significance of the varying location of EBP50 is not clear yet. Recent studies showed that phosphorylation, one of the major posttranslational modifications, strongly regulates the interaction of EBP50 with several proteins, like moesin and ezrin [8], [17]. Further investigations are necessary to clarify what is the function(s) of EBP50 in the nucleus and cytoplasm and what are the targeted proteins in these compartments.

The physiological significance of the phosphorylation of EBP50 was observed in wound healing as we found that the phospho-mimic mutant (S288D∶S310D) form of EBP50 significantly accelerated the wound healing of BPAEC. In vascular smooth muscle cells siRNA mediated depletion of EBP50 increased cell migration and induced cell morphology changes, moreover, binucleated cells were observed indicating the defect of cytokinesis [32]. *NHERF1/EBP50* gene mutations were identified in cancer cells [33], in addition knockdown of EBP50 increased cellular proliferation and migration of cancer cell lines suggesting that EBP50 acts as a tumor suppressor [34]. Our findings in normal cells underline the importance of EBP50 in cell division via the cyclin dependent kinase phosphorylation and PP2A dependent dephosphorylation of the protein.

Based on experiments with the aid of phosphatase inhibitors, it was suggested that the dephosphorylation of the Cdk1 (mitosis) -specific phosphoserine side chains in HeLa cells is mediated by PP1 or PP2A phosphatases, but not by PP2B [15]. Our attempts to verify the involvement of PP1 in the dephosphorylation of EBP50 in BPAEC have failed, since specific inhibitor (calyculin A, 2 nM) of PP1 had no effect on the phosphorylation state of EBP50 arrested in G2/M phase, moreover, immunoprecipitation experiments did not show protein-protein interaction between EBP50 and PP1. On the other hand, our results indicated that PP2A interacts and co-localizes with EBP50 in a cell cycle dependent manner. The holoenzyme form of PP2A is a heterotrimer that consists of a core dimer, composed of a scaffold (A) and a catalytic (C) subunit that associates with a variety of regulatory subunits (generally named B subunit) [19]. We did detect remarkable amount of A and C subunits in the immunoprecipitate of EBP50 and definite co-localization of the C subunit and EBP50 in the mitotic phase BPAEC. Furthermore, we identified Bα (also named as PR55) [19] with pull-down assays as the third subunit in the PP2A holoenzyme interacting with EBP50.

No PDZ domain was identified in the PP2A subunits and our results do not specify which subunit(s) of the PP2A heterotrimer is/are directly interacting with EBP50. A C-terminal motif was described in several peptides and proteins, which was shown to be responsible for binding to the PDZ domains in EBP50 [23], [35]. We compared the sequence of the four amino acids at the C-terminal of the PP2A subunits (LSLA for A, DYFL for C and DKVN for Bα subunit) with this DS/TxL motif. Although none of these tetrapeptides seem to be optimal, still we cannot exclude any subunit of PP2A as a potential binding partner for EBP50, as the results of others [23], [35] with peptide bonding indicate broad variations in the sequence.

The location change and the co-localization of EBP50 with PP2Ac during the course of mitosis were parallel, with the highest levels of co-localization during the phases from metaphase to early cytokinesis. We also observed that the ratio of dividing cells was higher in cells expressing the phospho-mimic mutant (S288D∶S310D) compared to those expressing the wild type EBP50. Since we could not detect the phosphorylated form of EBP50 in the lysate of interphase cells, these results suggests that the dephosphorylation of P-Ser^288^ and P-Ser^310^ is necessary for the mitotic exit and it is catalyzed by PP2A. Dephosphorylation of the Cdk1 substrates by PP1 and PP2A as an element of the exit from mitosis in vertebrate cells was described earlier [36], [37], [38].

Our results show that a protein thought to be a linker between the plasma membrane and cytoplasmic/cytoskeletal proteins may be involved in nuclear events as well. We provide evidences for cell cycle dependent localization and phosphorylation of EBP50, and its interaction with PP2A during mitosis. Our results indicate that EBP50 may have a significant role in the course of the cell cycle and that PP2A can be the phosphatase dephosphorylating P-Ser^288^ and P-Ser^310^ of EBP50 in BPAEC.

## Materials and Methods

Materials were obtained from the following sources: thymidine, nocodazole paraformaldehyde, dimethylsulfoxide, bovine serum albumin, Anti-V5 Agarose Affinity Gel antibody produced in mouse: Sigma (St Louis, MO); anti-PP1 catalytic subunit antibody: R&D System (Minneapolis, MN); anti-SLC9A3R1 antibody: Abgent (San Diego, CA); anti-PP2A B subunit (most specific for Bα) and anti-NHERF1(A310) antibodies, anti-rabbit IgG HRP-linked and anti-mouse IgG HRP-linked secondary antibodies: Cell Signaling Technology, Inc. (Beverly, MA); anti-PP2A B′ subunit, monoclonal anti-PP2Ac, monoclonal anti-β-tubulin antibodies: Upstate Biotechnology (Lake Placid, NY); anti-NHERF2 (C-2), anti-lamin A/C (H-110) antibodies: Santa Cruz Biotechnology, Inc. (Santa Cruz, CA); monoclonal anti-c-myc antibody: Zymed Laboratories (South San Francisco, CA); Protease Inhibitor Cocktail Set III EMD Biosciences (San Diego, CA); Alexa 488-, Alexa 546-conjugated secondary antibodies, Texas Red-phalloidin and ProLong Gold Antifade medium with DAPI: Molecular Probes (Eugene, OR), FuGENE® HD Transfection Reagent: Roche (South San Francisco, CA); pCMV-Myc and pcDNA3.1 V5-His vector: Clontech Laboratories, Inc. (Mountain View, CA); restriction enzymes, T4 DNA ligase, Phusion® High-Fidelity DNA Polymerase: Thermo Scientific, Inc. (Vantaa, Finland).

Substances for cell culturing were from Invitrogen Corporation (Carlsbad, CA). All other chemicals were obtained from Sigma (St Louis, MO).

### Cell cultures, cell synchronization, transfection

Bovine pulmonary artery endothelial cells (BPAEC) (culture line-CCL 209) were obtained frozen at passage 8 (American Type Tissue Culture Collection, Rockville, MD), and were utilized at passages 17–22. MCF7 (catalogue No: 86012803) cells were obtained frozen at passage 11 (European Collection of Cell Cultures, Salisbury, UK). Cells were maintained at 37°C in a humidified atmosphere of 5% CO_2_ and 95% air in MEM supplemented with 10% (v/v) fetal bovine serum (heat inactivated), 1% sodium pyruvate, 0.1 mM MEM non-essential amino acids solution. HeLa cells (catalogue No: 93021013) were obtained frozen at passage 4 (ECACC) and maintained in DMEM supplemented with 10% (v/v) FBS, 2 mM glutamine and 0.1 mM non-essential amino acids solution.

BPAEC were synchronized at G1/S phase using double thymidine block as follows. Cells were treated with 2 mM thymidine for 16 h. After this first thymidine block cells were released for 8 h and then treated with 2 mM thymidine for 16 h again. G2/M phase cells were obtained by 14–16 h treatment with 80 ng/ml nocodazole.

To express recombinant proteins 1 µg total DNA/3 µl FuGENE® HD reagent was mixed and added to the culture plates containing cells at ∼80% confluency according to the manufacturer's instruction. Cells were analyzed 24 h later.

### Cloning of EBP50

Total RNA was extracted from BPAEC using ZR RNA MicroPrep™ (Zymo Research Corporation, CA). cDNA was synthesized from 2 µg of total RNA using oligo-dT primer and M-MLV reverse transcriptase (Promega Corporation, USA). The coding region of wild-type EBP50 was amplified by PCR using the following primers (based on the sequence of SLC9A3R1, NM_001077852) containing *Xho*I and *Not*I restriction sites for subcloning: 5′-GC*CTCGAG*TTATGAGCGCGGACGCGG-3′ (forward) and 5′-ATAT*GCGGCCGC*TCAGAGGTTGCTGAAGAGTTC-3′ (reverse). The primers were synthesized by Integrated DNA Technologies (Coralville, IA). EBP50 S288D∶S310D mutant was created using oligonucleotides with the necessary mutant sequence. PCR products and pCMV-Myc vector were double digested with *Xho*I and *Not*I and ligated using 1∶3 vector∶insert molar ratio. The DNA sequences of the constructs were confirmed by sequencing (Clinical Genomics Center, MHSC, RCMM, University of Debrecen). One nucleotide deviance was detected (at position 192) that did not cause amino acid change.

### Immunofluorescence and microscopy

Cells were plated onto 0.2% gelatin coated glass coverslips and grown, washed once with 1× PBS (137 mM NaCl, 2.7 mM KCl, 4.3 mM Na_2_HPO_4_, 1.47 mM KH_2_PO_4_, pH 7.4) and fixed with 3.7% paraformaldehyde in 1× PBS for 15 min at room temperature. Between each step, the cells were rinsed three times with 1× PBS. The cells were permeabilized with 0.5% Triton X-100 in PBS at room temperature for 15 min, blocked with 2% BSA in PBS for 30 min at room temperature, and incubated with primary then with secondary antibodies diluted in blocking solution for 1 h at room temperature. Cover slips were rinsed and mounted in ProLong Gold Antifade medium.

Images were acquired with a Carl Zeiss Axioskope-20 microscope using Zeiss Plan-NEC FLUAR 63×1.25 NA oil immersion objective and Axiocam color camera (Zeiss, model 412-312). Confocal images were acquired with an Olympus Fluoview FV1000 confocal microscope using UPLSAPO 60×1.35 NA oil immersion objective on an inverted microscope (Olympus IX81) at 25°C. Images were processed using FV10-ASW v1.5 software and further processed with PhotoShop Imaging software.

Nonspecific binding of the secondary antibodies was checked in control experiments (not shown).

### Immunoprecipitation

BPAEC were grown in 100-mm tissue culture dishes and treated with thymidine or nocodazole, rinsed three times with 1× PBS and then collected and lysed with 600 µl of immunoprecipitation (IP) buffer (20 mM Tris HCl, pH 7.4, 150 mM NaCl, 2 mM EDTA, 2 mM sodium vanadate, 1% Nonidet P-40) containing protease inhibitors. The lysate was centrifuged with 10,000 *g* for 15 min at 4°C. To avoid nonspecific binding, the supernatants were precleared with 50 µl of protein G Sepharose (GE Healthcare, Piscataway, NJ) at 4°C for 3 h with end-over-end rotation. Protein G Sepharose was removed by centrifugation at 4°C for 10 min, and the supernatant was incubated with the appropriate volume of antibody at 4°C for 1 h and then with 50 µl of fresh protein G Sepharose at 4°C overnight with gentle rotation. The resin was washed three times with 300 µl of IP buffer and then resuspended in 150 µl of 1× SDS sample buffer, boiled, and microcentrifuged for 5 minutes. The supernatant was further analyzed by Western blot.

### Western blotting

Protein samples were separated by SDS-PAGE and transferred to 0.45 µm pore sized Hybond ECL Nitrocellulose Membrane (GE Healthcare, Piscataway, NJ). Western blots were imaged using an Alpha Innotech FluorChem® FC2 Imager.

### Subcellular fractionation

ProteoJET™ Cytoplasmic and Nuclear Protein Extraction Kit (Fermentas) were used for subcellular fractionation. Cells were collected in cell lysis buffer, containing 0.01 M DTT and protease inhibitor, vortexed and kept on ice for 10 min. Cytoplasmic fraction (CP1) was obtained by centrifugation at 500 *g* for 7 min and further cleaned by centrifugation at 13 000 *g* for 15 min at 4°C (CP2). Nuclear protein fraction (N) was obtained after washing the pellet from the first centrifugation two times with Nuclei washing buffer. The efficiency of fractionation was analyzed by immunoblotting using β-tubulin antibody as a cytoplasmic and lamin A/C antibody as a nuclear marker.

### GST pull-down assay

*Escherichia coli* BL21 (DE3) transformed with pGEX-4T-2 containing glutathione S-transferase (GST) or pGEX-4T-2 containing wild-type EBP50 coding DNA sequence fused with GST were induced with 1 mM IPTG and grown at 37°C with shaking for 3 h. Cells were harvested by centrifugation, sonicated in lysis buffer (50 mM Tris-HCl (pH 7.5), 0.1% Tween 20, 0.2% 2-mercaptoethanol, protease inhibitors) and proteins were isolated by affinity chromatography on glutathione Sepharose 4B (GE Healthcare, Piscataway, NJ) according to the manufacturer's protocol. BPAEC grown in 100-mm culture flasks were washed twice with 1× ice-cold PBS, scraped, and lysed in 600 µl lysis buffer. The lysates were incubated with GST or GST-EBP50 fusion proteins coupled to glutathione Sepharose beads for 2 h at 4°C. The beads were washed three times with 1× PBS then the GST fusion proteins were eluted with 10 mM glutathione and were tested by SDS-PAGE and confirmed by Western blot.

### Anti-V5 Agarose Affinity Gel

BPAEC grown in 6 well plates were transfected with pcDNA3.1 V5-His, pcDNA3.1 V5-His PP2A Bα, or pcDNA3.1 V5-His PP2A B′γ construct prepared in our laboratory. 24 h after transfection the cells were washed twice with 1× ice-cold PBS, scraped, and lysed in 600 µl lysis buffer (50 mM Tris-HCl (pH 7.5), 0.2% 2-mercaptoethanol and protease inhibitors). The cell lysates were sonicated then centrifuged at 10 000 *g* for 10 min at 4°C. The supernatant was added to 50 µl Anti-V5 Agarose conjugate and rotated for 5 h at 4°C. Beads were washed 3 times with PBS then boiled with 1× SDS buffer and analyzed by Western blot.

### *In vitro* wound healing assay

To study wound healing/cell migration ECIS (Electric cell-substrate impedance sensing) model Zθ, Applied BioPhysics Inc. (Troy, NY) was used that applies high electric field to make a well defined injury in a confluent cell monolayer and to screen the repopulation of this wounded area by noninvasive measurements [20], [39]. Wild type, mock, or mutant EBP50 transfected cells were seeded on type 8W10E arrays. After the cells achieved monolayer density (about 1000 Ω impedance), an alternate current of 5 mA at 60 kHz frequency was applied for 30 sec duration to establish wounds in the cell layer, which led to the death and detachment of cells present on the small active electrode, then the impedance was measured for 5 h. The impedance in each wounded well increased gradually, until it reached a maximum plateau value.

### Immunohistochemistry

Immunohistochemical staining on paraffin-embedded human skin sections (5 µm) was performed as follows. Samples were deparaffinized in xylene and rehydrated in decreasing concentrations (100, 95 and 70%) of ethanol followed by a 10 min incubation in PBS (pH 7.4). Sections were treated with 3% hydrogen peroxide in methanol for 15 min to block endogenous peroxidase activity and then rinsed briefly in PBS. Next the samples were subjected to antigen retrieval by heating for 2 min in a pressure cooker in 0.01 M sodium citrate buffer (pH 6.0). Nonspecific binding was blocked by incubating the slides for 60 min in PBS containing 1% BSA. EBP50 antibody was applied in a 1∶50 dilution in blocking solution O/N at 4°C and the negative control sections were incubated with blocking solution O/N at 4°C. After extensive washing (three times for 2 min) with PBS, samples were incubated with Alexa594 conjugated secondary antibody for 2 hours at room temperature in dark. Samples were rinsed for 5 min with PBS and mounted with ProLong® Gold Antifade Reagent with DAPI. Pictures were taken with a Zeiss Axioskope-20 microscope with 100×1.3 NA oil immersed objective.

### Statistical analysis

Pearson correlation coefficient was determined as described in [40]. Analysis of Variance on Ranks was performed on Pearson coefficients using SigmaStat. Statistical significance was determined at *P*<0.05 by Dunn's Method, multiple comparisons versus control group (group of interphase cells).

## Supporting Information

Figure S1 **Nuclear localization of EBP50 in BPAEC.** Immunofluorescence staining of confluent BPAEC was performed using anti-EBP50 (anti-NHERF1(A310) antibody, Cell Signaling Technology) (*a:* green) anti- primary antibodies. Actin microfilaments were stained with Texas Red conjugated phalloidin (*b:* red). *c* is merged image of *a* and *b*. Representative data of at least three independent experiments are shown. Scale bars: 100 µm.
(TIF)

Figure S2 **EBP50 is present in the nucleus of endothelial cells** ***in vivo*** **.** Immunofluorescent staining was performed on human skin sections using anti-EBP50 (anti-SLC9A3R1 antibody, Abgent) (*c:red*) primary antibody. Blood vessels were identified by morphological aspects using light microscope (*a, e*). Nuclei were stained with DAPI (*b, f: blue*). No non-specific binding of secondary antibody was detected in control experiment (*g*). *d* and *h* are merged images of *b–c* and *f–g*, respectively.
(TIF)
