# Supplementary figures and images for: Cell Cycle Dependent Association of EBP50 with Protein Phosphatase 2A in Endothelial Cells
Source: PLoS One. 2012 Apr 16;7(4):e35595. doi: 10.1371/journal.pone.0035595 (PMC3327649; doi:10.1371/journal.pone.0035595)

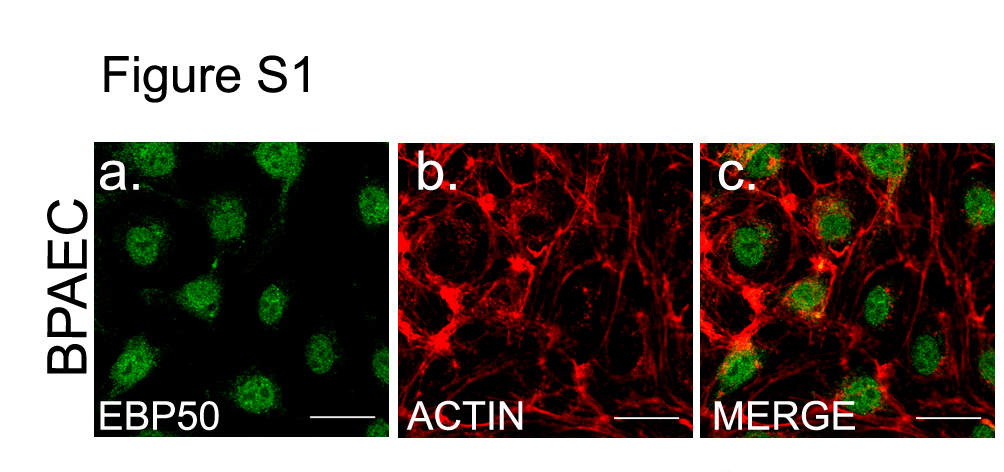

Supplement: Figure S1 — Nuclear localization of EBP50 in BPAEC. Immunofluorescence staining of confluent BPAEC was performed using anti-EBP50 (anti-NHERF1(A310) antibody, Cell Signaling Technology) (a: green) anti- primary antibodies. Actin microfilaments were stained with Texas Red conjugated phalloidin (b: red). c is merged image of a and b. Representative data of at least three independent experiments are shown. Scale bars: 100 µm. (TIF) [file pone.0035595.s001.TIF]

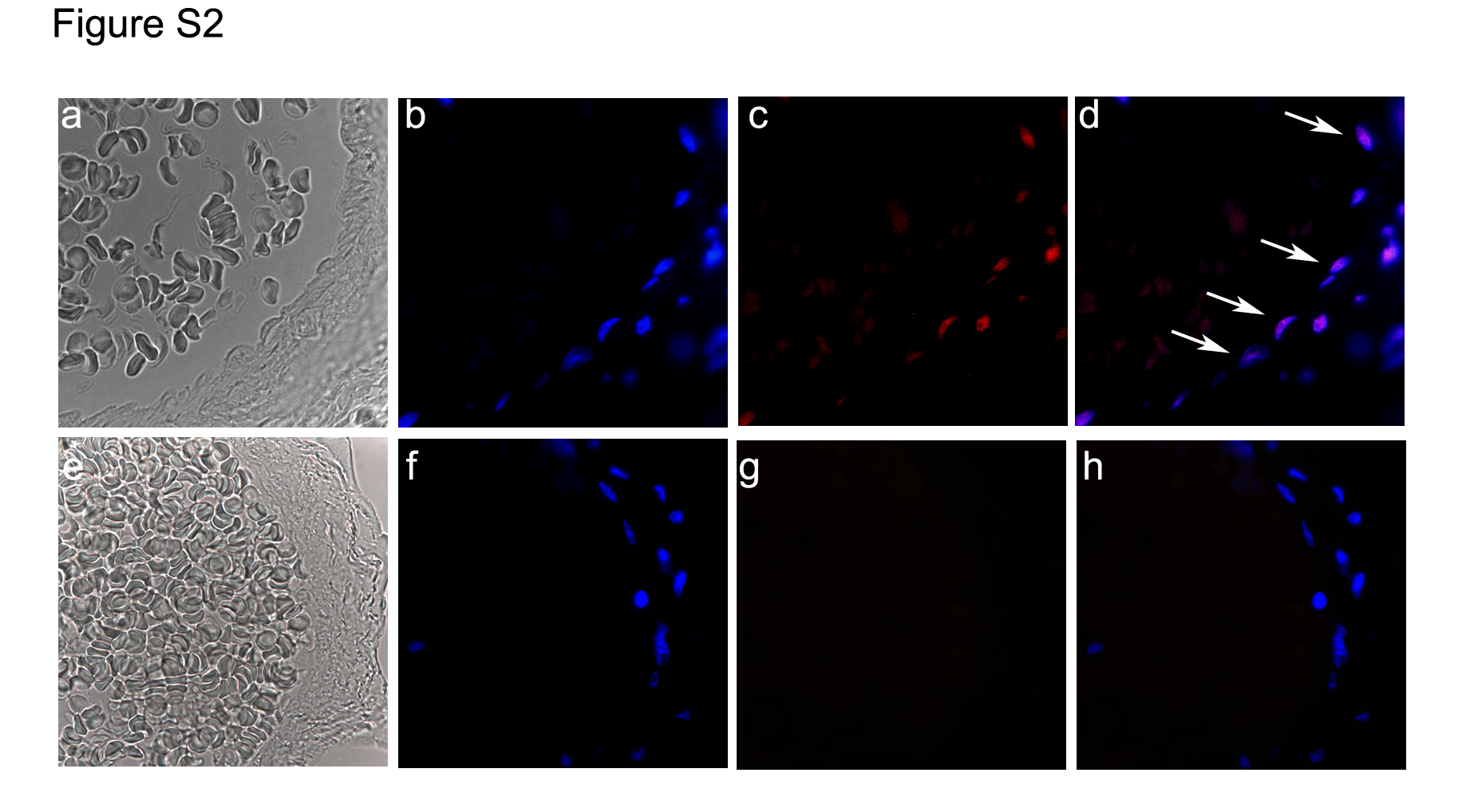

Supplement: Figure S2 — EBP50 is present in the nucleus of endothelial cells in vivo . Immunofluorescent staining was performed on human skin sections using anti-EBP50 (anti-SLC9A3R1 antibody, Abgent) (c:red) primary antibody. Blood vessels were identified by morphological aspects using light microscope (a, e). Nuclei were stained with DAPI (b, f: blue). No non-specific binding of secondary antibody was detected in control experiment (g). d and h are merged images of b–c and f–g, respectively. (TIF) [file pone.0035595.s002.TIF]
